# Comparative transcriptome analysis of SARS-CoV-2, SARS-CoV, MERS-CoV, and HCoV-229E identifying potential IFN/ISGs targets for inhibiting virus replication

**DOI:** 10.3389/fmed.2023.1267903

**Published:** 2023-12-08

**Authors:** Yuzhuang Liu, Tianyi Lu, Cuidan Li, Xiaotong Wang, Fei Chen, Liya Yue, Chunlai Jiang

**Affiliations:** ^1^National Engineering Laboratory for AIDS Vaccine, School of Life Sciences, Jilin University, Changchun, China; ^2^CAS Key Laboratory of Genome Sciences and Information, Beijing Institute of Genomics, Chinese Academy of Sciences and China National Center for Bioinformation, Beijing, China; ^3^Beijing Institute of Genomics, University of Chinese Academy of Sciences, Beijing, China; ^4^Beijing Key Laboratory of Genome and Precision Medicine Technologies, Beijing, China; ^5^Key Laboratory for Molecular Enzymology and Engineering of the Ministry of Education, School of Life Sciences, Jilin University, Changchun, China

**Keywords:** SARS-CoV-2, SARS-CoV, MERS-CoV, IFN response, ISGs, pathogenesis

## Abstract

**Introduction:**

Since its outbreak in December 2019, SARS-CoV-2 has spread rapidly across the world, posing significant threats and challenges to global public health. SARS-CoV-2, together with SARS-CoV and MERS-CoV, is a highly pathogenic coronavirus that contributes to fatal pneumonia. Understanding the similarities and differences at the transcriptome level between SARS-CoV-2, SARS-CoV, as well as MERS-CoV is critical for developing effective strategies against these viruses.

**Methods:**

In this article, we comparatively analyzed publicly available transcriptome data of human cell lines infected with highly pathogenic SARS-CoV-2, SARS-CoV, MERS-CoV, and lowly pathogenic HCoV-229E. The host gene expression profiles during human coronavirus (HCoV) infections were generated, and the pathways and biological functions involved in immune responses, antiviral efficacy, and organ damage were intensively elucidated.

**Results:**

Our results indicated that SARS-CoV-2 induced a stronger immune response versus the other two highly pathogenic HCoVs. Specifically, SARS-CoV-2 induced robust type I and type III IFN responses, marked by higher upregulation of type I and type III IFNs, as well as numerous interferon-stimulated genes (ISGs). Further Ingenuity Pathway Analysis (IPA) revealed the important role of ISGs for impeding SARS-CoV-2 infection, and the interferon/ISGs could be potential targets for therapeutic interventions. Moreover, our results uncovered that SARS-CoV-2 infection was linked to an enhanced risk of multi-organ toxicity in contrast to the other two highly pathogenic HCoVs.

**Discussion:**

These findings provided valuable insights into the pathogenic mechanism of SARS-CoV-2, which showed a similar pathological feature but a lower fatality rate compared to SARS-CoV and MERS-CoV.

## 1 Introduction

Over the past 20 years, highly pathogenic human coronaviruses (HCoVs), consisting of SARS-CoV-2, SARS-CoV, and MERS-CoV, have caused three life-threatening epidemics (1). SARS-CoV emerged in 2002–2003 and rapidly spread throughout Asia, infecting approximately 8,000 people and causing 774 deaths, with a mortality of about 9.6% (2). MERS-CoV, which was discovered in the Middle East in 2012, infected 2,519 people, of whom 866 died, representing a mortality of about 34% (3). SARS-CoV-2, which emerged in December 2019, has resulted in a global pandemic with immense damage to human health and the economy, causing approximately 761 million infections and 7.9 million deaths by March 2023 (4). The mortality rate of SARS-CoV-2 varied between countries and populations, with an overall pooled mortality of 5.6% (5). Although SARS-CoV-2 has a lower fatality rate than the other two, it still poses a significant threat to human health due to its rapid and elusive mutation and the possibility of reinfection after vaccination (6, 7).

SARS-CoV, MERS-CoV, and SARS-CoV-2 have similar clinical symptoms, such as cough, fever, and brachypnoea (8). In severe cases, all three diseases can progress to fatal acute respiratory distress syndrome and multiple organ damage. Although the SARS-CoV-2-induced COVID-19 disease shares consistent clinical features with SARS and MERS, it is less lethal than them. In addition, compared to these highly pathogenic HCoVs, the prevalent strains of coronavirus (HCoV-OC43, HCoV-229E, HCoV-HKU, and HCoV-NL63) generally lead to mild respiratory illnesses like the common cold (9, 10). The reason for this varying pathogenicity of different HCoVs is still a subject under research. Limited evidence suggests that some factors may contribute to their differences in pathogenicity, such as the ability to replicate efficiently in humans, the immune escape ability, and the damage caused by promoting excessive inflammation.

Cell invasion and replication are essential to virus infection, these processes are usually mediated by the interaction between viral S proteins and cellular receptors (11). SARS-CoV-2 encoded longer S proteins compared with SARS-CoV and MERS-CoV (12). ACE2 is the common cellular receptor for SARS-CoV and SARS-CoV-2. However, the affinity of ACE2 to SARS-CoV-2 is 10–20 times higher than that to SARS-CoV (13, 14). HCoVs also evolve different strategies to escape host immunity. Human respiratory viruses spread along the nasopharyngeal tract, which partly avoids the innate immune cells (15). In severe infections with SARS-CoV-2, the innate immunity is diminished. However, monocytes and macrophages are recruited to the infected tissues, which induce excessive inflammation (16). In addition to the decline of lymphocytes during SARS-CoV infection, the virus interferes with some protective immune intracellular pathways, thereby preventing the production of IFN-1 and the activation of T cells (17). MERS-CoV encodes ORF4a, which acts as an antagonist of IFN to escape the host innate immunity (18).

The immune system is the major line of defense against intrusive viruses. Previous studies revealed the expression of some genes associated with the immune response during HCoV infections. Most of the over-expressed gene products in acute-phase sera of SARS patients were involved in immunity, such as *IFN-*γ, *TGF-*β, *IL-6*, *IL-8*, and *IL-18* (19, 20). Similarly, the serum levels of *IFN-*α, *CXCL10*, *IL-6*, *IL-8*, and *CCL5* were elevated in the patients with MERS-CoV, especially in those with severe MERS-CoV (21, 22). A similar cytokine profile of *IFN-*γ, *TNF-*α, *IL-2*, *IL-6*, *IL-10*, *IL-1*β, and *GM-CSF* was also observed in COVID-19 patients (23–25). Most of these upregulated genes during highly pathogenic HCoV infections are inflammatory cytokines, which suggests a dysregulated inflammatory response.

Research on the host responses to HCoVs with different pathogenicity could help develop effective interventions and therapies against the ongoing COVID-19 and future pandemics from the host perspective. Transcriptome analysis provides a powerful tool for studying host immune dynamics, and it also facilitates the identification of the regulated genes and signaling pathways during infections. A meta-analysis of publicly available transcriptome data from cell lines identified the common regulated pathways during infection with SARS-CoV-2, SARS-CoV, and MERS-CoV (26). These findings revealed that enhancing glutathione metabolism may reduce the severity of the SARS-CoV-2 infection. Additionally, a study on the transcriptional response to SARS-CoV, MERS-CoV, SARS-CoV-2, IAV, HPIV3, and RSV indicated that diminished innate antiviral defenses and overproduction of inflammatory cytokines are the driving features of COVID-19 (27). However, these studies mostly focused on the three highly pathogenic viruses or other additional pathogenic respiratory viruses. The differences in host responses induced by different HCoVs with varying pathogenicity are still not fully elucidated.

In the present study, we analyzed the transcriptome data from four publicly available datasets of human cell lines. We identified and compared the differences in gene expression and pathway regulation during highly pathogenic SARS-CoV-2, SARS-CoV, MERS-CoV, and lowly pathogenic HCoV-229E infections. The obtained results contribute to comprehending the pathogenesis of these coronaviruses and controlling any future HCoV outbreaks.

## 2 Materials and methods

### 2.1 Data collection and processing

We employed a search of the Gene Expression Omnibus (GEO) database to identify transcriptome datasets related to the seven HCoV infections in human respiratory cell lines. Of these, we selected four datasets related to SARS-CoV, SARS-CoV-2, MERS-CoV, and HCoV-229E for further analysis and listed them in Supplementary Table 1. Specifically, we focused only on the transcriptome data of the Calu3 and MRC-5 cell lines, as well as their corresponding mock controls, at the 24-h time point after infection with the four viruses in each dataset. The transcriptome data of PBMC samples from COVID-19 patients and corresponding healthy controls was obtained from GSE152418 dataset. All RNA-Seq data from our article were accessible through the NCBI GEO with the accession numbers GSE147507 (27), GSE56189, GSE148729 (28), GSE155986 (29), and GSE152418. The SARS-CoV-2 strains used in GSE147507 (USA-WA1/2020) and GSE148729 (Patient isolate, BetaCoV/Munich/BavPat1/2020| EPI_ISL_406862) were early original strains.

The FASTQ data from all these datasets was downloaded with the SRA toolkit and quality-controlled with fastp (30). The “fastq-dump” command was then employed to extract the fastq sequence information. Subsequently, we employed the “split-files” function to split the sequencing data, followed by applying a standardized quality control using the “fastp” tool with the parameter “-q 25 -u 30 thread = 5” to remove reads with substandard base quality. The human reference genome GRCh38.84 was used for alignment through HISAT2 (v2.2.0), and the mitochondrial genes were removed. Reads with alignment confidence scores exceeding 20 were extracted using “-F 4 -q 20,” resulting in the generation of the gene expression profiles required for downstream analyses. Gene expression levels of the sequencing data were quantified using the DESeq2 R package (version 1.18.1) and visualized through boxplots (Supplementary Figure 1A). Only genes with a count per million (CPM) greater than 1 in at least half of the samples were retained. The VennDiagram package in R (version 1.6.20) was implemented to plot Venn diagrams to visualize the relationship between genes in each infection group (Supplementary Figure 1B). The removal of the batch effects resulting from different links, such as methods, cell lines, and experimental designs, was achieved by the “removeBatchEffect” function of the limma R package (version 3.48.3). Finally, the results were checked through the fviz_pca_ind function and the plotted principal component analysis (PCA) diagrams (Supplementary Figure 1C).

### 2.2 Data analysis

**FIGURE 1:**
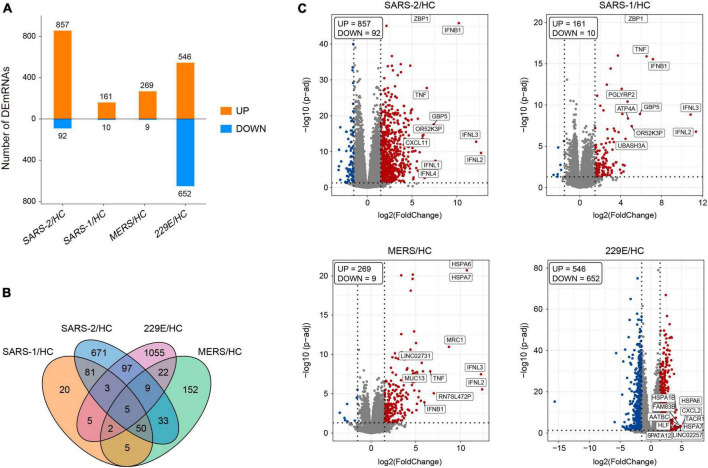
Differentially expressed genes analysis of the four HCoV groups *in vitro* compared with the uninfected cells. **(A)** Bar plot of the differentially expressed mRNAs compared with the uninfected cells in each group. **(B)** Venn diagram of differentially expressed mRNA in the four HCoV-infected groups. **(C)** Volcano plots of the four HCoV groups based on the DEGs. The upregulated DEGs with the top 10 log2FC are shown.

The edgeR package (version 3.34.0) was executed for analyzing the differences in mRNA expression among the four HCoVs groups and the mock controls. The adjusted *p*-values of 0.05 together with absolute log2 (fold change) >1.5 were used as thresholds for identifying the differentially expressed genes (DEGs), and the up- and downregulated DEGs in each group were identified (Figure 1A). The count of the unique and common DEGs in each group was completed using the R package VennDiagram (Figure 1B). Volcano plots of the DEGs in each group were plotted using the ggplot2 R package (version 3.3.5), with the top 10 upregulated DEGs marked.

### 2.3 Pathway and functional enrichment analysis

**FIGURE 2:**
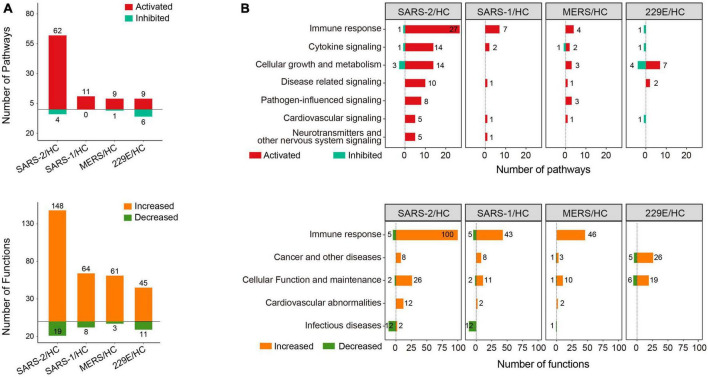
Pathway and function enrichment analysis upon HCoV infections based on IPA. **(A)** Quantity of the pathways and functional terms enriched in the HCoV-infected groups. **(B)** Classifications of the pathways and functional terms based on functional characteristics. For IPA analysis, –log (*p*-value) >1.3 and absolute *Z*-score >2 were set as the thresholds to identify the significantly up- or downregulated pathways and functional terms.

Function and pathway enrichment analysis of the DEGs were implemented with the software Ingenuity Pathway Analysis (IPA, Ingenuity Systems, Inc.) (31). For the IPA analysis, −log10 (*p*-value) >1.3 together with absolute *Z*-score >2 was set as the thresholds to identify the significantly up- or downregulated pathways and functional terms. The enrichment analysis results were visualized using positive and negative bar graphs (Figure 2A). The pathway and function terms were grouped into different categories based on functional characteristics (Figure 2B). Bubble plots of the enriched terms were generated with the ggplot2 R package. The heatmap of cytokine related DEGs was drawn using the R function pheatmap. The Cytoscape software was employed to exhibit the relationship between the genes and enriched functions. Tox function analysis was also conducted using IPA software.

## 3 Results

### 3.1 Significant upregulation of DEGs during highly pathogenic HCoV infections

In this study, we analyzed publicly available transcriptome data from cells infected with different coronaviruses (SARS-CoV-2, SARS-CoV, MERS-CoV, and HCoV-229E) (Supplementary Table 1). The results of PCA revealed distinct mRNA expression profiles among the four groups of infected and mock-infected cells (Supplementary Figure 1C). In particular, we observed that the SARS-CoV-2 cluster exhibited a closer distance to the SARS-CoV and MERS-CoV clusters than to the HCoV-229E cluster. This finding indicated marked variations in gene expression between highly pathogenic and lowly pathogenic HCoVs.

To further explore the differences in gene expression patterns among HCoVs, we identified the DEGs among the four groups of infected cells. In total, 949, 171, 278, and 1,198 DEGs were observed in SARS-CoV-2, SARS-CoV, MERS-CoV, as well as HCoV-229E, respectively (Figure 1A). Notably, we observed that a large proportion of DEGs (70.7 and 88.1%) were specifically identified in the SARS-CoV-2 and lowly pathogenic HCoV-229E group, respectively (Figure 1B). These findings indicated significant differences between highly and lowly pathogenic HCoVs. Besides these, the results also revealed that the majority of DEGs were significantly upregulated in the highly pathogenic HCoV-infected groups, particularly in the SARS-CoV-2 group, where 90.3% of the 949 DEGs showed an upregulation (Figure 1A). These observations suggested that infected cells may mount a resistance response to highly pathogenic HCoVs by upregulating the expression of certain genes.

To identify the key genes involved in the host response to each virus, we analyzed the top 10 upregulated DEGs in each group (Figure 1C). In the highly pathogenic SARS-CoV-2 group, 8 out of the top 10 upregulated DEGs were interferon cytokine genes (*IFNL2*, *IFNL3*, *IFNB1*, *IFNL1*, and *IFNL4*) and IFN-stimulated genes (*GBP5*, *TNF*, and *ZBP1*), indicating a robust interferon response in SARS-CoV-2 infection. Similarly, in the SARS-CoV group, 6 out of the top 10 upregulated DEGs were interferon cytokine genes (IFNs) and ISGs, including *IFNL2*, *IFNL3*, *IFNB1*, *GBP5*, *TNF*, and *ZBP1*. In the MERS-CoV group, 4 out of the top 10 upregulated DEGs were IFNs and ISGs, including *IFNL2*, *IFNL3*, *IFNB1*, and *TNF*. Additionally, *MRC1*, which is related to antigen recognition (32), was also among the top upregulated DEGs in the MERS-CoV group. In the HCoV-229E group, the top upregulated DEGs, such as *FAM83B*, *AATBC*, *Linc02257*, and *SPATA12*, were mainly related to cell proliferation and migration (33–36) and DNA oxidative damage (37).

### 3.2 SARS-CoV-2 induces stronger immune responses than SARS-CoV and MERS-CoV

To gain insights into the molecular mechanisms underlying the pathogenicity of different HCoVs and to identify enriched pathways and functional categories, we conducted an IPA analysis. The analysis revealed a total of 66, 11, 10, and 15 pathways, as well as 167, 72, 64, and 56 functional terms, in the highly pathogenic SARS-CoV-2, SARS-CoV, MERS-CoV, and lowly pathogenic HCoV-229E groups, respectively (Figure 2A). Notably, the majority of enriched pathways and functions were significantly activated across all four groups [*Z*-score ≥2 and −log (*p*-value) >1.3], with the SARS-CoV-2 group displaying the highest number of activated terms (Figure 2A).

To provide a comprehensive overview of the enriched pathways and functional terms, we have grouped them based on their functional characteristics (Figure 2B). The results showed that both the top-ranked pathway or function term category in all three highly pathogenic HCoV groups was “Immune response.” Specifically, 27, 7, and 4 pathways and 100, 43, and 46 functions associated with “Immune response,” were significantly activated across all three highly pathogenic HCoV groups (Figure 2B). In contrast, only one significantly inhibited immune response-related pathway (“Interferon Signaling”) was discovered in the lowly pathogenic HCoV-229E. Moreover, none of the immune-related function terms were significantly activated or inhibited [*Z*-score ≥2 and −log (*p*-value) >1.3]. Our findings suggested that highly pathogenic HCoVs induced a robust immune response, while lowly pathogenic HCoVs might not activate extra immune responses or elicit a weaker immune response.

**FIGURE 3:**
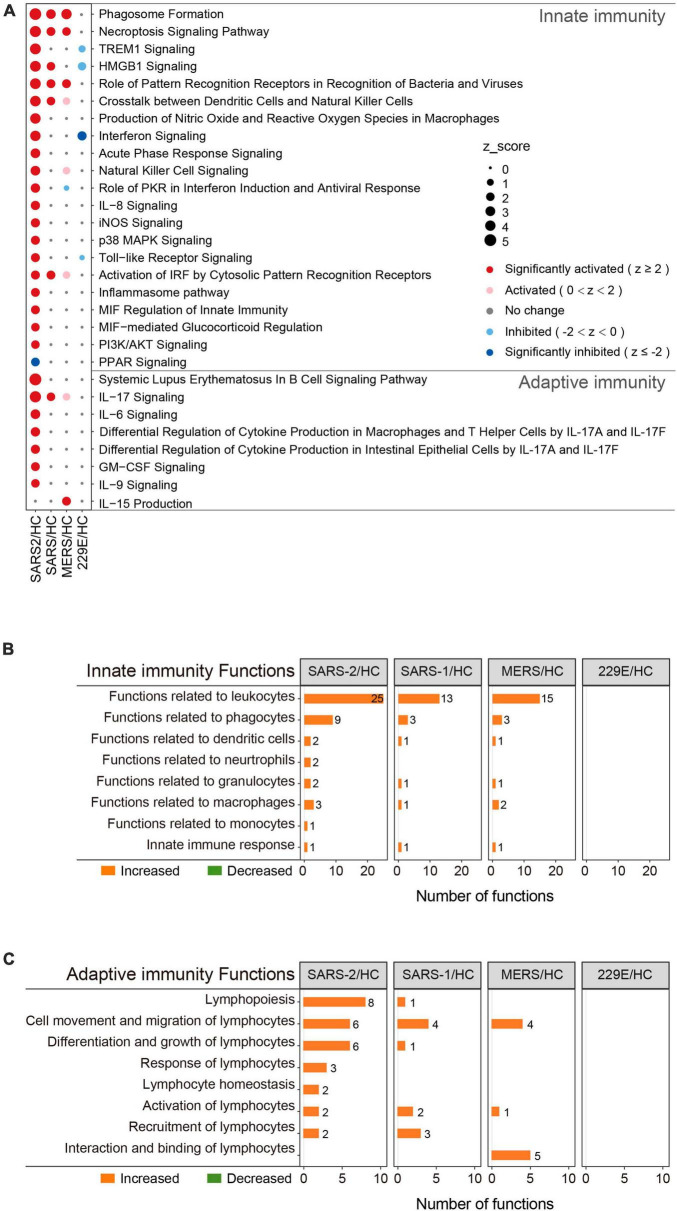
Immunity-related pathway and functional enrichment analysis of the DEGs using IPA. **(A)** Bubble plot of the pathways related to innate and adaptive immunity that were enriched in each group. The size of the dots represents the *Z*-score for each term. Bubble plots of the functional terms related to innate immunity **(B)** and adaptive **(C)** immunity.

To determine the precise immunological response of cells to the highly pathogenic HCoVs, we intensively investigated the innate and adaptive immune responses of infected cells (Figure 3). For innate immunity, the SARS-CoV-2 infection significantly activated 20 out of the 21 enriched pathways and all of the 45 enriched functions (Figures 3A, B). Of these, 14 pathways and 28 functions were unique to the SARS-CoV-2 infection, such as “Toll-like Receptor Signaling” (*Z* = 2.333), “Production of Nitric Oxide and Reactive Oxygen Species in Macrophages” (*Z* = 3.606), “iNOS Signaling” (*Z* = 2.449), “Natural Killer Cell Signaling” (*Z* = 2.887), “Interferon Signaling” (*Z* = 3.5), and “Role of PKR in Interferon Induction and Antiviral Response” (*Z* = 2.673). In contrast, the SARS-CoV and MERS-CoV groups exhibited fewer enriched innate immune pathways and functions (Figures 3A, B). Only three common innate immune-associated pathways, including “Phagosome Formation,” “Role of Pattern Recognition Receptors in Recognition of Bacteria and Viruses,” and “Necroptosis Signaling Pathway,” as well as 10 innate immune-associated functions, were significantly activated in all three highly pathogenic HCoV groups (Supplementary Figure 2A). Our results indicated that the innate immune response triggered by SARS-CoV-2 infection was distinct from that of SARS-CoV and MERS-CoV and may be potentially capable of inducing stronger innate immune responses than the other two.

This was also true for adaptive immunity in response to highly pathogenic HCoV infections (Figures 3A, C). The SARS-CoV-2 group exhibited 7 significantly activated adaptive immunity pathways and 29 functions, including 6 pathways and 23 functions that were specific to the SARS-CoV-2 infection. Interestingly, three out of the seven adaptive immunity pathways were related to IL-17 (Figure 3A). In contrast, the SARS-CoV and MERS-CoV groups displayed less enrichment in adaptive immune pathways and functions, with only “IL-17 signaling” (*Z* = 2) and “IL-15 production” (*Z* = 2.236) being significantly activated in the SARS-CoV and MERS-CoV groups, respectively. Furthermore, there were no common adaptive immunity-associated pathways among the three groups, and only three adaptive immunity-associated functions were significantly activated in all three groups, which were “Cell movement of lymphocytes,” “Lymphocyte migration,” and “T cell migration” (Supplementary Figure 2B). Collectively, these results uncovered that SARS-CoV-2 infection evoked more specific and robust innate and adaptive immune responses than SARS-CoV and MERS-CoV.

### 3.3 Specific upregulation of chemokines and interleukins during SARS-CoV-2 infection

**FIGURE 4:**
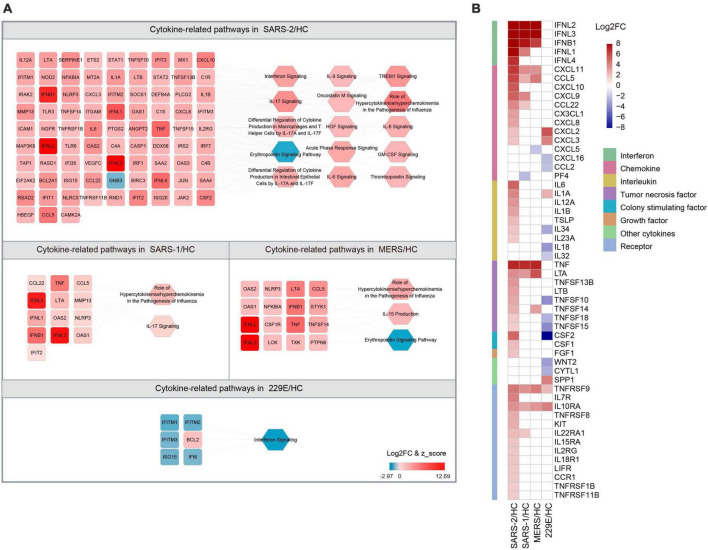
Expression of cytokine-related DEGs in the four HCoV groups. **(A)** Networks of the cytokine genes and cytokine-related pathways in each HCoV-infected group. Up- and downregulated genes and pathways are presented in red and blue, respectively. **(B)** Heatmap of the DEGs encoding cytokines and cytokine receptors in each group.

The COVID-19 severity is linked to an excessive inflammatory response characterized by the generation of a substantial amount of pro-inflammatory cytokines, a phenomenon known as the cytokine storm (38). To explore the differences in cytokine responses among the highly pathogenic SARS-CoV-2, SARS-CoV, MERS-CoV, and the lowly pathogenic HCoV-229E, we investigated the cytokines and cytokine-related pathways in each group (Figure 4).

Our results unveiled that SARS-CoV-2 infection significantly activated 14 cytokine-related pathways, whereas only two cytokine-related pathways were activated in the SARS-CoV and MERS-CoV group, respectively (Figure 4A). The “Role of Hypercytokinemia/hyperchemokinemia in the Pathogenesis of Influenza” pathway displayed the highest enrichment among all three highly pathogenic HCoV groups. The *Z*-scores for this pathway in the SARS-CoV-2, SARS-CoV, and MERS-CoV groups were 5.568, 3.162, and 2.828, respectively. Notably, no activated cytokine-related pathways and only one inhibited pathway, “Interferon signaling” (*Z* = −2.449), were identified in the lowly pathogenic HCoV-229E group.

To further explore the cytokine profiles during infection with these HCoVs, we analyzed the expression levels of the 41 cytokines and 13 cytokine receptor genes that were significantly regulated during infections (Figure 4B). Our analysis signified that the SARS-CoV-2 group exhibited significantly higher cytokine and cytokine receptor gene expression levels than the other three HCoV groups. Among these, the upregulated chemokines and interleukins were the most common cytokine genes in the SARS-CoV-2 group. Specifically, three chemokine genes (*CXCL10*, *CXCL8*, and *CX3CL1*) and five interleukin genes (*IL-6*, *IL-12A*, *IL-1B*, *IL-23A*, and *TSLP*) were found to be specifically upregulated. In contrast, no interleukin-related genes were observed to be upregulated in the SARS-CoV or MERS-CoV group. Furthermore, three interleukin genes were found to be downregulated in the HCoV-229E group, with *CSF2* (log2FC = −15.644) exhibiting the most significant downregulation among the interleukin genes. These findings suggested that chemokines and interleukins might play a pivotal role in the pathogenesis of SARS-CoV-2 infection.

### 3.4 Strong IFN/ISG response responsible for the inhibition of SARS-CoV-2 replication

The IFNs and ISGs, which are mainly involved in innate immunity, have been identified as being highly upregulated in response to infection with highly pathogenic HCoVs (Figure 1C). To further investigate this phenomenon, we analyzed the expression levels of IFNs and ISGs in the four HCoV groups. Our results demonstrated that IFNs were significantly upregulated in cells infected with the highly pathogenic SARS-CoV-2, SARS-CoV, and MERS-CoV, whereas no significant upregulation was observed in cells upon infection with the lowly pathogenic HCoV-229E (Figure 4B). Of particular interest, the top three upregulated IFNs in highly pathogenic HCoVs were type III *IFNL2*, *IFNL3*, and type I *IFNB1*. Notably, our analysis revealed that type III *IFNL1* was commonly upregulated in both SARS-CoV-2 and SARS-CoV groups, while type III *IFNL4* was uniquely identified in the SARS-CoV-2 group. Furthermore, we noted that the “Interferon signaling” pathway was significantly activated (*Z* = 3.5) in the SARS-CoV-2 group, whereas this pathway was significantly inhibited (*Z* = −2.449) in the lowly pathogenic HCoV-229E (Figure 4A). These results highlighted the critical role of interferon in the host’s immune response to highly pathogenic HCoV infections.

**FIGURE 5:**
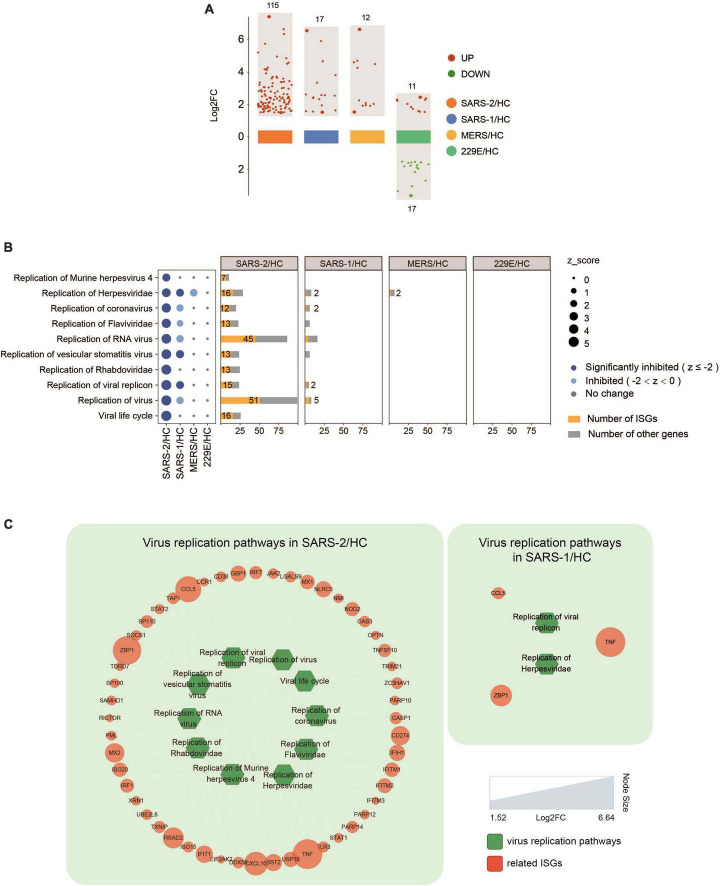
Expression of ISGs in the four HCoV groups. **(A)** Scatter plot of the ISGs in the four HCoV-infected groups. Up- and downregulated ISGs are presented in red and green, respectively. **(B)** Virus replication-related functional terms and their corresponding DEGs. ISGs and other genes enriched in each virus replication-related term are presented in yellow and gray, respectively. **(C)** Networks of the significantly downregulated virus replication-related functions and the correlated ISGs. The size of the circles represents the log2FC for each ISG.

Further analysis of the expression levels of 628 known ISGs (39) in the four HCoV groups revealed that SARS-CoV-2 exhibited significantly higher levels of ISG expression than the other three HCoVs (Figure 5 and Supplementary Table 2). Specifically, we observed that 115 ISGs were distinctly upregulated in cells with the SARS-CoV-2 infection, implying a robust interferon response (Figure 5A). Notably, 95 of these ISGs (82.6%) were uniquely upregulated in the SARS-CoV-2 group (Supplementary Table 2). Our investigation further revealed an upregulation of several ISGs with known antiviral functions, including *IFIT*, *IFITM*, *RSAD2*, *ZNFX1*, *TRIM21*, *ISG15*, and *ISG20* (40–45). Moreover, we observed an upregulation of GBP genes (*GBP3*, *GBP4*, *GBP5*, and *GBP6*), which have been demonstrated to modulate extensive innate immune responses against various pathogens (46). In addition, the upregulation of genes that potentiated IFN signaling was also observed, including *TAP1*, *STAT1*, and *XAF1* (47–50). In contrast to SARS-CoV-2, infections with SARS-CoV and MERS-CoV induced significantly fewer ISGs (17 and 12, respectively), and infection with the lowly pathogenic HCoV-229E resulted in the downregulation of 17 out of 28 ISGs (Figure 5A).

Inhibition of virus replication has been demonstrated in many ISGs. To investigate the effectiveness of ISGs in inhibiting virus replication, we conducted a functional analysis that identified 52 ISGs that were significantly enriched in ten functional terms associated with virus replication (Figures 5B, C). These functional terms displayed significant inhibition in the highly pathogenic SARS-CoV-2 group, including “Viral life cycle” (*Z* = −4.491), “Replication of virus” (*Z* = −4.392), and “Replication of coronavirus” (*Z* = −3.517). Furthermore, it was found that more than half of the DEGs (52–64%) enriched in each functional term related to virus replication were ISGs (Figure 5B). This indicated a strong IFN/ISG response that contributed to inhibiting SARS-CoV-2 replication. In contrast, only three functional terms related to virus replication were inhibited in the SARS-CoV group (Figure 5C), including “Replication of Herpesviridae” (*Z* = −2.214), “Replication of vesicular stomatitis virus” (*Z* = −2.425), and “Replication of viral replicon” (*Z* = −2.236), which included three ISGs (*TNF*, *ZBP1*, and *CCL5*). Interestingly, no such virus replication-related functions were found in the MERS-CoV group.

To further investigate the putative role of ISGs in shaping the clinical course of COVID-19, we conducted an analysis using publicly accessible PBMC transcriptome data sourced from the GSE152418 dataset. This dataset comprises PBMC samples collected from a cohort of 15 COVID-19 patients exhibiting various degrees of symptom severity, with categorizations ranging from moderate, severe, to critical, alongside a control group consisting of 17 healthy individuals. We identified a total of 107 ISGs, demonstrating distinctive expression profiles across the spectrum of COVID-19 symptom severity, with marked upregulation in moderate cases and conspicuous downregulation in severe patients, particularly those requiring ICU admission (Supplementary Figure 3 and Supplementary Table 3). Of notable significance, among these ISGs, we found that 13 ISGs exhibited a significant upregulation in patients with moderate COVID-19 symptoms, while 17 ISGs displayed a significant downregulation in patients necessitating ICU admission. This observed pattern in ISG expression harmoniously reinforces the conclusions drawn from our prior investigation of lung cell lines. Notably, these findings suggested the potential role of insufficient ISG expression and its subsequent suppression as plausible contributory factors to the manifestation of more severe clinical symptoms in COVID-19 patients.

Overall, our findings demonstrated that SARS-CoV-2 infection induced a distinctive and strong interferon response characterized by the upregulation of multiple ISGs with antiviral functions. These results provided valuable insights into the pathological characteristics of SARS-CoV-2, which showed a relatively milder pathogenesis and a lower fatality rate in contrast to SARS-CoV and MERS-CoV.

### 3.5 Multi-organ toxicity of SARS-CoV-2 infection

**FIGURE 6:**
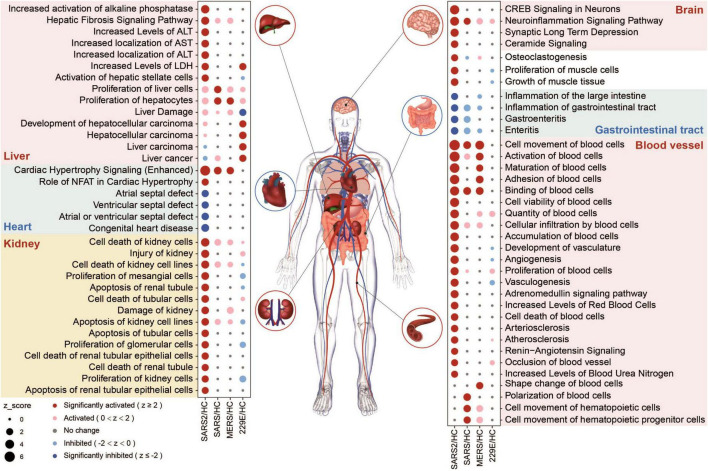
Organ-related functional enrichment analysis based on IPA predicted the multi-organ damage in different HCoV infections.

We evaluated the potential multi-tissue injury using IPA tox-function analyses. Our results revealed that SARS-CoV-2 infection significantly upregulated multi-organ damage related functions, particularly in the liver, kidney, and vasculature (Figure 6). In contrast, fewer relevant terms were significantly enriched in the other three HCoV groups.

In the SARS-CoV-2 group, four functions associated with the angiocarpy system were significantly upregulated (Figure 6). These activated functions were all associated with elevated blood pressure. In contrast, the other four downregulated functions in this group were associated with congenital heart disease, including atrial and ventricular septal defects (Figure 6). These findings suggested that SARS-CoV-2 infection can increase blood pressure and reduce the risk of congenital heart malformations, which may be realized through the effect of ACE2 on the renin-angiotensin system (RAS) (51). In general, our findings unveiled that the SARS-CoV-2 infection had a more pronounced effect on multi-organ toxicity than other HCoV infections.

## 4 Discussion

SARS-CoV-2, SARS-CoV, and MERS-CoV are all members of the Coronaviridae family and have been associated with severe respiratory illnesses in humans. The pathological manifestations of COVID-19 share similarities with those seen in SARS and MERS. However, SARS-CoV-2 has a comparatively milder pathogenesis and a lower fatality rate in contrast to SARS-CoV and MERS-CoV. A comprehensive study of the factors that lead to the lower virulence of SARS-CoV-2 is essential for developing effective interventions and therapies against the disease.

In this work, we revealed that SARS-CoV-2 elicited a more significant immune response modulation, particularly in the interferon response, than SARS-CoV, MERS-CoV, and HCoV-229E (Figure 3). IFNs function in the immune response to viral infections by activating the immune cells and inducing the production of antiviral proteins. Our results suggest that SARS-CoV-2 can induce robust type I and type III IFN responses, characterized by a notable upregulation of both type I and type III IFNs, along with the induction of numerous ISGs. The type I interferon gene *IFNB1* and type III interferon cytokine genes (*IFNL2* and *IFNL3*), as well as 11 ISGs, were significantly upregulated in the highly pathogenic SARS-CoV-2, SARS-CoV, and MERS groups (Figure 4B and Supplementary Table 2), indicating common antiviral mechanisms of highly pathogenic coronaviruses. The type III interferon gene *IFNL1* was significantly upregulated in both SARS-CoV-2 and SARS-CoV groups but not following MERS-CoV infection. IFNL1 can inhibit viral replication in infected cells (52). In addition to its antiviral activity, IFNL1 also plays a part in modulating the immune response, helping to regulate inflammation, and preventing tissue damage (53). Besides, the upregulation of *IFNL4* was observed only in SARS-CoV-2 infection (Figure 4B). IFNL4 is a member of the human type III IFNs (54) and plays a critical role in antiviral immunity. IFNL4 activates the JAK/STAT pathway, leading to the expression of ISGs (55). It is reported that IFNL4 has stronger therapeutic effects in reducing coronavirus infection with higher ISG induction compared to other type III IFNs (56). Given the importance of the IFN response in the control of viral infections, IFN (*IFNL1* and *IFNL4*) targeted therapies for COVID-19 are currently being developed.

Interferon-stimulated genes that impeded virus replication during the four HCoV infections were also identified. Specifically, we have identified 52 and 3 ISGs that were significantly enriched in functional terms associated with virus replication in the SARS-CoV-2 and SARS-CoV groups, respectively. None of these ISGs were identified in the MERS-CoV infection. These observations provided valuable insights into the comparatively milder pathogenesis and lower fatality rate of SARS-CoV-2, in contrast to SARS-CoV and MERS-CoV. Moreover, these ISGs could be treated as potential therapeutic targets against highly pathogenic HCoV infections. Indeed, we have identified 13 of these ISGs as drug targets (Supplementary Table 2). Among these, the JAK2 inhibitors, Ruxolitinib and Tofacitinib, had been used to treat COVID-19 and cytokine release syndrome (57, 58). Additionally, Rintatolimod, a TLR3 agonist, had been suggested for treating post-COVID syndrome (59). Drugs and vaccines for SARS-CoV-2 infection are being developed (60–62). These ISG agonists identified in this study hold promise for treating COVID-19 patients, while further studies are warranted to validate their efficacy and safety in larger clinical trials.

The interferon response is the first defense for the host against virus infections. HCoVs have evolved certain proteins that can inhibit IFN responses to evade host immunity, which may result in different IFN responses after infections with different HCoVs. The significant differences in the ability to activate interferon-related pathways of different HCoVs may reflect the mutational differences among them. ORF6 is an important virulence factor of HCoVs that can inhibit IFN response. Previous studies found that the inhibitory efficiency of SARS-CoV-2 ORF6 on IFN response is lower than that of SARS-CoV ORF6 (63). Replacement of SARS-CoV ORF6 with the full-length gene of SARS-CoV-2 ORF6 or deletion of SARS-CoV ORF6 by stop codon results in decreased replication efficiency of SARS-CoV, while the ability of ORF6 to antagonize IFN response is weakened. Papain-like protease (PLpro) is another crucial IFN antagonist found in HCoVs. PLpro can inhibit the activation of IFN response by suppressing IRF3 through its deubiquitinating activity and de-ISGylation activity (64). Compared to SARS-CoV, SARS-CoV-2 PLpro has a higher buried interface size, a conserved catalytic triad (C111, H278, and D293), and a lower number of interacting residues in ubiquitin with PLpro. These differences might contribute to a slightly lower deubiquitinating activity in SARS-CoV-2 Plpro. Both SARS-CoV and SARS-CoV-2 NSP1 proteins can inhibit IFN response. However, Lacasse et al. (65) discovered that NSP2 of SARS-CoV-2 can activate the NF-κB pathway and the IFNβ promoter. They also found that NSP2 of SARS-CoV-2 partially counteracts the IFN inhibitory activity of NSP1. The phenomenon of genetic mutations altering the ability of the virus to induce IFN responses is not unique to HCoVs but is also present in other viruses, such as the Zika virus. The A188V mutation on the NS1 protein of Zika virus can reduce the phosphorylation of TBK1, thereby leading to a decreased expression level of IFNβ (66). Therefore, it is speculated that SARS-CoV-2 significantly activates IFN response, which may be attributed to genetic mutations in certain genes of SARS-CoV-2. These genetic mutations enhance the ability of certain proteins to activate IFN response or weaken the inhibitory capacity of certain proteins against IFN, as compared to SARS-CoV.

The immune response is a double-edged sword. On the one hand, an effective immune response can limit viral replication and clear the virus, thereby reducing morbidity and mortality. On the other hand, an exaggerated and dysregulated immune response can result in a cytokine storm, leading to tissue damage, multi-organ dysfunction, and even death. SARS-CoV-2 infection has been demonstrated to contribute to a cytokine storm, which is linked to COVID-19 severity and is also a very important determinant of mortality of COVID-19 (67). In contrast, SARS-CoV and MERS-CoV infections are associated with a lower incidence of cytokine storms (68). The findings of our article revealed that SARS-CoV-2 induced a higher expression of multiple cytokine genes and activated more cytokine-related pathways than SARS-CoV, MERS-CoV, and HCoV-229E (Figures 4A, B), and the cytokines *IL-6*, *CXCL10*, and *CSF2* might serve as pivotal determinants in triggering the onset of a cytokine storm in SARS-CoV-2. This finding was consistent with previous studies on the cytokine storm in COVID-19 (23, 69), where a plethora of cytokines were produced following SARS-CoV-2 infection. Recent research has highlighted the importance of the IL-6/CXCL10/macrophage axis in driving the initiation and maintenance of the cytokine storm (70). IL-6 could activate the JAK/STAT pathway, resulting in the large production of cytokines, especially chemokines (70–72). These cytokines induce the local enrichment of *CSF2*, leading to further JAK/STAT pathway activation in the inflamed tissues (73). *CSF2* and chemokines, especially *CXCL10*, form a channel for invading immune cells and promote the recruitment of many immune cells in the lung tissues, which readily induce tissue damage. This characteristic vicious cycle of the SARS-CoV-2 infection-evoked cytokine storm was not observed in HCoV-229E-infected cells, where the downregulation of *CSF2* might prevent the activation and recruitment of innate immune cells, avoid hyperactivation of the immune response, inflammation, and tissue damage.

These identified cytokines, such as *IL-6*, *CXCL10*, and *CSF2*, could be potential therapeutic targets for COVID-19 damage management. Blocking the IL-6 pathway has been demonstrated to be an effective approach for reversing pulmonary failure and reducing mortality in COVID-19 (74–76). CXCL10 may exert functions in the development of COVID-19, as described in research on the role of the CXCL10-CXCR3 axis in the pathogenesis of COVID-19 (77). Anti-CSF2 receptor monoclonal antibodies have been used to improve clinical symptoms in COVID-19 patients with severe pulmonary disease (78). Further research is needed to investigate the mechanism of differential regulation of cytokines, especially *IL-6*, *CXCL10*, and *CSF2*, between highly pathogenic HCoVs and common circulating HCoVs.

This study aimed to investigate the differential transcriptome responses induced by the four HCoVs. Nevertheless, it is important to recognize the limitations of our study. Firstly, the lack of available high-throughput sequencing data from patients infected with SARS-CoV during the 2003 outbreak prevented their inclusion in our analysis. Additionally, due to the rapid control of the epidemic, limited transcriptome data were available from these patients. Besides, the relatively mild symptoms and low mortality rate associated with common HCoVs (HCoV-HKU, HCoV-229E, HCoV-OC43, and HCoV-NL63) resulted in a paucity of transcriptome data for analysis. Furthermore, validation of the identified targets by *in vitro* experiments is impossible owing to laboratory constraints. In spite of these limitations, we believe that our analysis provides valuable insights into the different pathogenesis of highly and lowly pathogenic HCoVs. The identification of key IFNs and ISGs in our study may be beneficial for the future development of treatments for COVID-19 together with other highly pathogenic coronaviruses.

## Data availability statement

The datasets presented in this study can be found in online repositories. The names of the repository/repositories and accession number(s) can be found in the article/Supplementary material.

## Author contributions

YL: Conceptualization, Investigation, Methodology, Software, Writing – original draft, Writing – review and editing. TL: Methodology, Software, Writing – original draft. CL: Methodology, Writing – original draft. XW: Writing – review and editing. FC: Writing – review and editing. LY: Conceptualization, Methodology, Supervision, Writing – review and editing. CJ: Conceptualization, Supervision, Writing – review and editing.
