# Supplementary material for: Comparative transcriptome analysis of SARS-CoV-2, SARS-CoV, MERS-CoV, and HCoV-229E identifying potential IFN/ISGs targets for inhibiting virus replication
Source: Front Med (Lausanne). 2023 Dec 8;10:1267903. doi: 10.3389/fmed.2023.1267903 (PMC10739311; doi:10.3389/fmed.2023.1267903)
Supplement: Supplementary file 1 [file Data_Sheet_1.docx]

Supplementary Material

Comparative transcriptome analysis of SARS-CoV-2, SARS-CoV, MERS-CoV, and 229E identifying potential IFN/ISGs targets for inhibiting virus replication

Yuzhuang Liu^1^, Tianyi Lu^2, 3^, Cuidan Li^2^, Xiaotong Wang^2^, Fei Chen^2, 3, 4^, Liya Yue^2*^, and Chunlai Jiang^1, 5*^

^1^National Engineering Laboratory for AIDS Vaccine, School of Life Sciences, Jilin University, Changchun, China

^2^CAS Key Laboratory of Genome Sciences & Information, Beijing Institute of Genomics, Chinese Academy of Sciences and China National Center for Bioinformation, Beijing, China

^3^University of Chinese Academy of Sciences, Beijing, China

^4^Beijing Key Laboratory of Genome and Precision Medicine Technologies, Beijing, China

^5^Key Laboratory for Molecular Enzymology and Engineering of the Ministry of Education, School of Life Sciences, Jilin University, Changchun, China

*** Correspondence:**Liya Yue, yuely@big.ac.cn; Chunlai Jiang, jiangcl@jlu.edu.cn

# Supplementary Figures and Tables

## Supplementary Figures

**
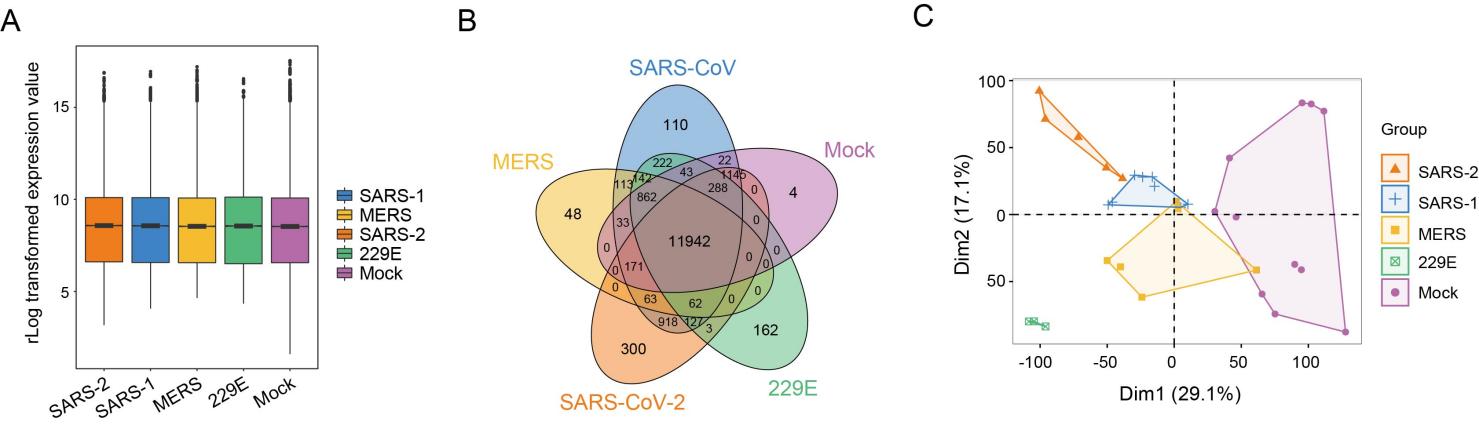
**

**Supplementary Figure 1.** Transcriptome information of human lung cell lines after infection with different HCoVs. **(A)** Expression levels of mRNA in the four hCoV groups and their corresponding mock controls. **(B)** Venn diagram of mRNA expression across the five groups. **(C)** PCA plot of the five groups after removing the batch effects.


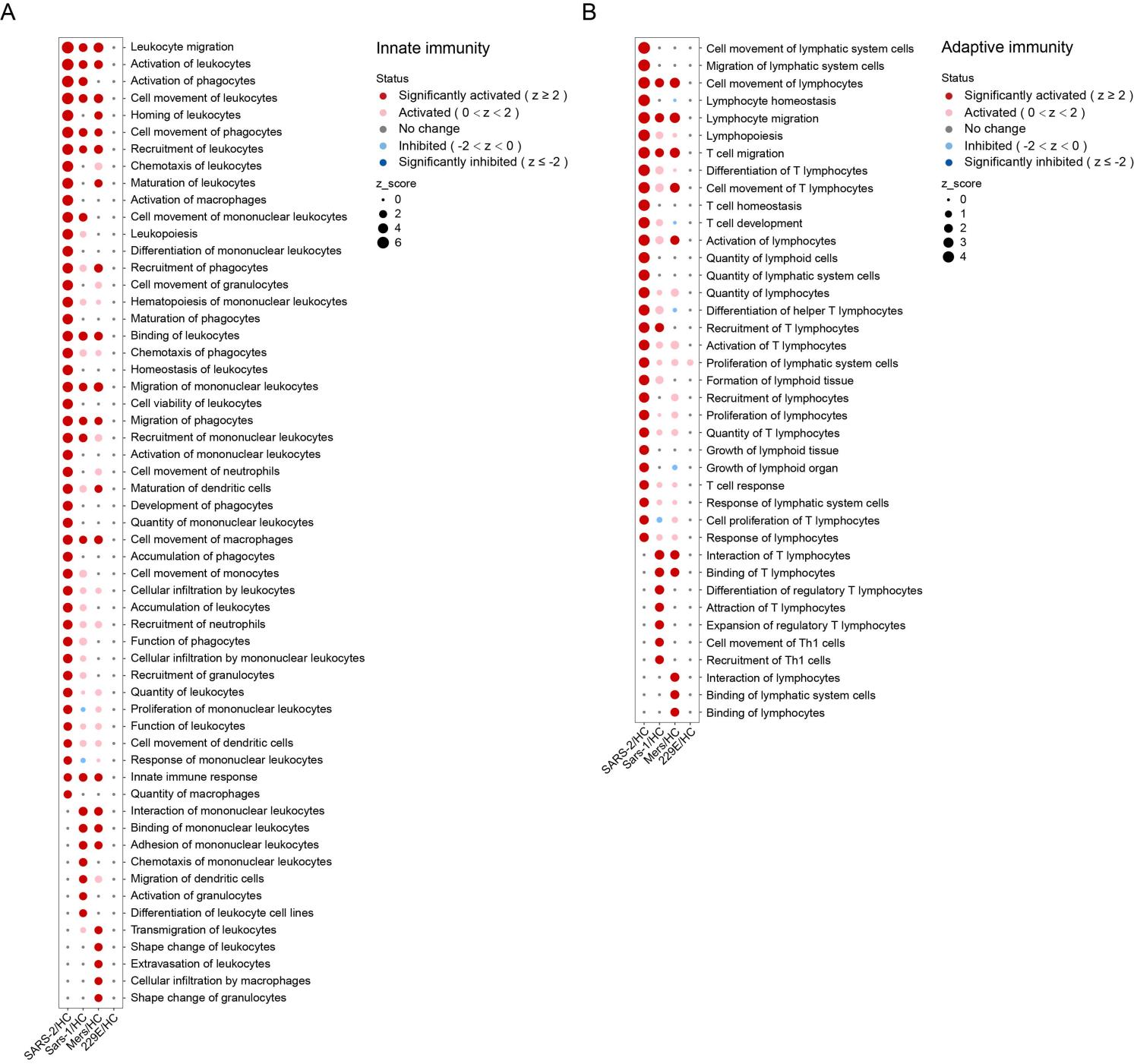


**Supplementary Figure 2.** Bubble plots of the functional terms involved in innate **(A)** and adaptive **(B)** immunity in HCoV-infected groups. The size of the dots represents the z-score for each term.


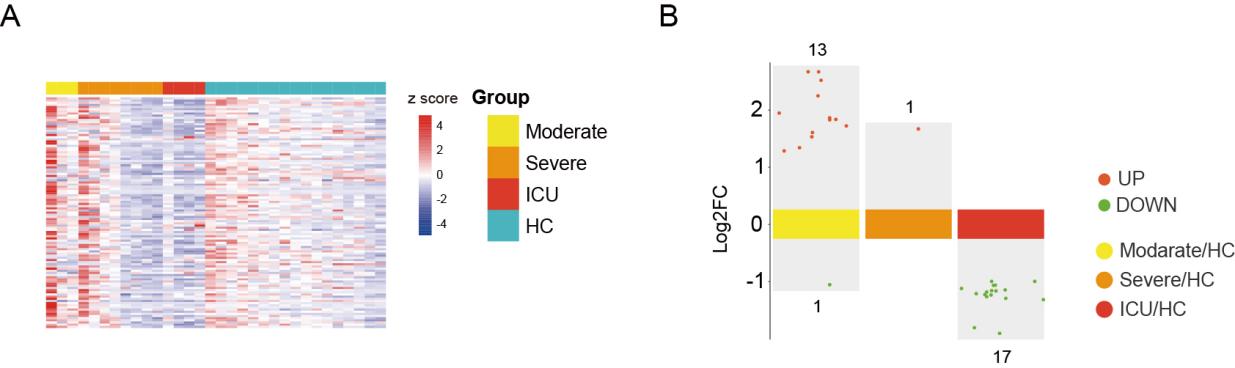


**Supplementary Figure 3.** **(A)** The ISGs expression levels in COVID-19 patients with varying symptom severity and healthy controls. **(B)** Scatter plot of the ISGs in the three groups. Up- and downregulated ISGs are presented in red and green, respectively.

## Supplementary Tables

| **Virus** | **Time** | **Number of samples** | **Cell type** | **Accession number** |
| --- | --- | --- | --- | --- |
| SARS-CoV-2 | 24h | 2 | Calu3 | GSE148729 |
| SARS-CoV-2 | 24h | 3 | Calu3 | GSE147507 |
| SARS-CoV | 24h | 2 | Calu3 | GSE148729 |
| SARS-CoV | 24h | 6 | MRC-5 | GSE56189 |
| MERS-CoV | 24h | 6 | MRC-5 | GSE56189 |
| HCoV-229E | 24h | 3 | MRC-5 | GSE155986 |
| Mock | 24h | 3 | MRC-5 | GSE56189 |
| Mock | 24h | 2 | Calu3 | GSE148729 |
| Mock | 24h | 3 | Calu3 | GSE147507 |
| Mock | 24h | 3 | MRC-5 | GSE155986 |

**Supplementary Table 1.** Datasets of the human coronaviruses used in this study.
